# Person-centered care content in medicine, occupational therapy, nursing, and physiotherapy education programs

**DOI:** 10.1186/s12909-022-03502-8

**Published:** 2022-06-24

**Authors:** Catarina Wallengren, Håkan Billig, Ida Björkman, Inger Ekman, Caroline Feldthusen, Irma Lindström Kjellberg, Mari Lundberg

**Affiliations:** 1 University of Gothenburg Centre for Person-Centred Care (GPCC), Sahlgrenska Academy, University of Gothenburg, P.O. Box 457, 40530 Gothenburg, Sweden; 2 Department of Learning and Leadership for Health Care Professionals, Institute of Health and Care Sciences, The Sahlgrenska Academy, University of Gothenburg, P.O. Box 457, 40530 Gothenburg, Sweden; 3 Institute of Neuroscience and Physiology, University of Gothenburg, Gothenburg, Sweden; 4 Department of Life Context and Health Promotion, Institute of Health and Care Sciences, Sahlgrenska Academy, University of Gothenburg, Gothenburg, Sweden; 5 Institute of Health and Care Sciences, Sahlgrenska Academy, University of Gothenburg, Gothenburg, Sweden; 6 Medicine/Geriatrics, Sahlgrenska University Hospital/Östra, Gothenburg, Sweden; 7 Division of Physiotherapy, Department of Health and Rehabilitation, Institute of Neuroscience and Physiology, University of Gothenburg, Gothenburg, Sweden; 8 Department of Health Promoting Science, Sophiahemmet University, Stockholm, Sweden

**Keywords:** Descriptive study design, Higher education institutions, PCC, National study programs, Local program syllabuses, Local course syllabuses

## Abstract

**Background:**

Although person-centered care (PCC) ensures high-quality care for patients, studies have shown that it is unevenly applied in clinical practice. The extent to which future health care providers are currently offered education in PCC at their universities is unclear. We aimed to clarify the PCC content offered to students as a basis for their understanding by exploring the PCC content of Swedish national study programs in medicine, nursing, occupational therapy, and physiotherapy.

**Methods:**

Using a qualitative document analysis design, we sampled the steering documents from all higher education institutions (*n* = 48) with accreditation in medicine (*n* = 7), nursing (*n* = 25), occupational therapy (*n* = 8), or physiotherapy (*n* = 8) at a single time point. All national study programs (*n* = 4), local program syllabuses (*n* = 48), and local course syllabuses (*n* = 799) were reviewed using a 10-item protocol.

**Results:**

We found no content related to PCC in the steering documents at the national level. At the local level, however, signs of PCC were identified in local program syllabuses and local course syllabuses. Seven of the 48 local program syllabuses (15%) included PCC in their intended learning outcomes. Eight of the 799 local course syllabuses (1%) contained course titles that included the phrase ‘person-centered care,’ and another 101 listed 142 intended learning outcomes referring to PCC. A total of 21 terms connected to PCC were found, and the term ‘person-centered care’ was most commonly used in the nursing programs and least commonly in the medical programs.

**Conclusions:**

There is a broad range in how the national study programs in Sweden have incorporated PCC. The implementation has been driven by a bottom-up strategy. A deliberate and standardized strategy is needed to ensure full implementation of PCC into clinical curricula in higher education.

## Background

Patients, caregivers, and the World Health Organization have all called for a shift towards person-centered care (PCC) [1, 2], moving away from care based on New Public Management principles to care based on mutual respect and collaboration between the patient and the clinician. In Canada [3], Eastern Mediterranean Region [4], England [5], Indonesia [6], Sweden [7], and USA [8], discussions are in progress about what implementing PCC may mean in the context of their respective health care systems.

Several frameworks exist for PCC [8–11], including a recently presented European standard: ‘Patient involvement in health care—Minimum requirements for PCC to ensure quality improvement in PCC’ [12]. Even if these frameworks differ somewhat, they all share the same foundational principle that the patient is not a disease or diagnosis, but a person who has the ability and resources to express will, needs, and desires and who wants and can take responsibility for her/his own life. Several meta-analyses have pointed out convincing evidence for the effect of PCC [13–16] on a variety of outcomes, such as improved blood pressure and psychological health. In addition, there are multiple randomized controlled trials that have shown an effect on and reduction of health care costs [17, 18].

A successful implementation strategy for PCC requires that several actors from different health and social care sectors work together in a unified approach to mutually drive forward change [6, 19, 20]. This requirement also implicates educational institutions as important enablers of PCC given their roles in training future health and social care workers [6]. In several countries, PCC is being introduced into the university and college education systems, including Australia [21], Scotland [22], and Sweden [23]. Some studies have explored PCC in medical and nursing undergraduate curricula [24–26]. However, to the best of our knowledge, no studies have examined the emphasis on or inclusion of PCC concomitantly across multiple national education programs in different clinical areas.

## Methods

### Aim

The overall aim was to explore the PCC content in four Swedish national study programs: medicine, nursing, occupational therapy, and physiotherapy.

### Design

This study used a qualitative document analysis design [27].

### Setting

Swedish national study programs are funded publicly, and the education system is governed legally by the Swedish Higher Education Act [28] and Higher Education Ordinance [29]. In 2007, Swedish higher education was adapted to the European Bologna process [30]. For this study we will refer to three different levels: level I, national study programs; level II, local program syllabuses; and level III, local course syllabuses. The national study programs (level I) are to have local program syllabuses (level II) and be accompanied by local course syllabuses (level III). These steering documents must contain information about included courses and requirements for special eligibility. The mandatory information in the local course syllabuses is a description of the level, the number of higher education credits (HECs) earned with course completion, intended learning outcomes, special eligibility, and forms of assessment. Optional information includes associated literature and compulsory elements. An independent government agency, the Swedish Higher Education Authority, regularly assesses the quality of Swedish higher education [31].

### Description of context

In spring 2020, the Swedish national study program (level I) in medicine comprised 330 HECs, which corresponds to 11 semesters of full-time studies provided at seven higher education institutions (HEIs). The national study programs in nursing, occupational therapy, and physiotherapy (level 1) comprise 180 HECs or six semesters of full-time studies. The national nursing program is offered at 25 HEIs and the national programs in occupational therapy and physiotherapy at 8 institutions each. In spring 2020, a total of 10,895 students were admitted to these four national study programs: 2155 to the program in medicine, 7218 to the program in nursing, 693 to the program in occupational therapy, and 829 to the program in physical therapy [32].

### Data extraction

To extract data from national steering documents, such as national study programs (level 1), local program syllabues (level II), and local course syllabuses (level III), a protocol was developed in three phases.

### Phase 1. Setting up a steering committee

A steering committee consisting of three researchers (CF, IB, CW). In terms of the researchers’ prior understanding, all had previous research experience with PCC and were affiliated with University of Gothenburg Centre for Person-Centred Care (GPCC). Moreover, they all had previous teaching experience and knowledge and understanding of steering documents at the three levels. Their task was to develop a protocol to examine the steering documents.

### Phase 2. Developing a protocol

A crucial step in developing a protocol was to a priori agree upon the conceptual definitions of PCC to include in the study. PCC is described within several frameworks and, even though the authors adhered to the description of PCC by Ekman et al. [9], our research goal was to apply a broad understanding of PCC in the search process. This broad approach was chosen for two reasons. First, information in the literature suggests that the choice of terms affects the professional approach and execution in clinical practice [33–35]. Second, there are multiple descriptions of PCC [36]. As a result, the research group concluded that documents that have content referring to actors (person, patient, client) and are linked to context (care, rehabilitation), actions (listening, documenting), and relationships (co-creation, approach) should be accepted as having content equivalent to PCC as described in previous work [8–12].

A syllabus governs and controls the content and learning outcomes of education programs; thus, it highlights the values of a specific society [37, 38]. According to the Swedish National Agency for Higher Education (UKÄ) [31], the national study program (level I), local program syllabuses (level II), and local course syllabuses (level III) must account for which courses are included in the program and which content and intended learning outcomes are in each level. According to Swedish law [28, 29], each steering document needs to have a short description of its contents. This content consists of subject content on which the teaching must focus. The intended learning outcomes should be classified into three categories: knowledge/comprehensibility, skills/ability, and judgment/attitude. In cases of unclear classification of the intended learning outcomes, Bloom’s taxonomy [39] could be used to guide the examination. We chose to use Bloom’s taxonomy in unclear situations because of its history as a fruitful tool for developing and evaluating levels of knowledge in documents used in higher education [40, 41].

Thus, the steering group wanted to examine the total number of intended learning outcomes referring to actors and linked to context, actions, and relationships in the national study plan (level I), a total number of included local program syllabuses (level II) and local course syllabuses (level III), intended learning outcomes, courses, and course titles. Furthermore, the total distribution of the intended learning outcomes by semester in the study programs and how they were distributed around what students should have achieved at the end of the course regarding knowledge/comprehension and skills judgment/attitude was calculated. Finally, there was an interest in examining the contents describing PCC and variants of this term.

In the first level of the protocol constructed based on the above choices, one question was crafted to examine the national study plans for the four programs. In the second level of the protocol, four questions were crafted to examine local program syllabuses (*n* = 48), and in the third level of the protocol five questions to examine the local course syllabuses (*n* = 799; Table 1).

**Table 1:** Overview of the three levels examined and questions asked

Level	Questions
I. National study plan	1. What is the total number of intended learning outcomes containing content referring to actors and linked to context, actions, and relationships?
II. Local program syllabuses	2. What is the total number of local program syllabuses? 3. What is the total number of intended learning outcomes that contain content referring to actors and linked to context, actions, and relationships? 4. What is the total number of courses included in the program syllabuses? 5. What is the total number of course titles with content referring to actors and linked to context, actions, and relationships?
III. Local course syllabuses	6. What is the total number of examined local course syllabuses? 7. What is the total number of intended learning outcomes with content referring to actors and linked to context, actions, and relationships? 8. How are intended learning outcomes, with content that refers to actors and linked to context, actions and relationships, distributed between different semesters of the educational programs? 9. How is the total number of intended learning outcomes distributed under the headings knowledge/comprehensibility, skills/ability, or judgment/attitude? 10. What is the total number of terms that describe content referring to actors and linked to context, actions, and relationships?

### Phase 3. Evaluation of steering documents

The appointed researchers (CF, IB, CW) retrieved data on the national study programs and syllabuses at all three levels from the university and college websites between January and May 2020. Thereafter, the researchers separately examined the national study program (level I), local program syllabuses (level II), and local course syllabuses (level III) using the protocol described in Table 1. The researchers checked the results of the protocol review and any differences discussed until consensus was reached [42].

### Data analysis

According to previous authors [27], document analysis is intended to identify, select, evaluate, and synthesize the content in the documents, which in this study involves content referring to actors and linked to context, actions, and relationships. We used content analysis [43, 44] to analyze the steering documents in three steps: reading of documents, coding and categorizing, and calculating frequencies and percentages.

### Reading of documents

The steering documents were read (CF, IB, CW) several times to achieve overall familiarity and a picture of their manifest content (i.e., phenomenon – content referring to actors and linked to context, actions, and relationships).

### Coding and building categories

Meaning units (words, sentences, paragraphs) were selected by the researchers (CF, IB, CW) using a deductive approach. PCC context was searched for in the the local program (level II) and local course syllabuses (level III) (questions 2 and 6), as well as intended learning outcomes (questions 1, 3, and 7), courses (question 4), course titles (question 5), semesters (question 8), headings of learning levels (question 9), and terms connected to actors, context, actions, and relationships (question 10). Identified meaning units were then extracted, condensed, and labeled with a code. Finally, the codes were sorted and abstracted into categories. Deductive coding was used to assign an appropriate heading for an intended learning outcome (question 9). The unassigned headings were coded into a code map based on Bloom’s taxonomy [39]. Unassigned headings were sorted into three categories: knowledge/comprehensibility, skills/ability, and judgment/attitude. Finally, all of the codes were sorted and abstracted into categories. Thus, in the deductive analyses, each word or sentence was coded, condensed, and grouped to describe the explicit content. Any differences between the researchers (CF, IB, CW) in the data interpretation were discussed until consensus was reached [42–44].

### Calculating frequencies and percentages

Frequencies and percentages were calculated to describe the total number of local program and course syllabuses (questions 2 and 6), intended learning outcomes (question 1, 3, and 7), courses (question 4), course titles (question 5), semesters (question 8), headings of learning levels (question 9), and terms connected to actors, context, actions, and relationships (question 10) [27].

### Trustworthiness

To ensure trustworthiness, several actions were planned a priori. Credibility was ensured by the fact that all researchers (CF, IB, CW) who collected data have experience reading and interpreting steering documents in higher education (reflexivity). The data collection period lasted for 5 months (prolonged engagement). All researchers in the study participated in the discussion on how the results should be interpreted and understood (member checking). To account for the study's dependability, there was a description of how the steering documents were retrieved, identified, analyzed, and described (investigator triangulation). Confirmability was secured by describing how the study protocol was created and what phenomenon (i.e., content referring to actors and linked to context, actions, and relationships) we looked for in the steering documents (audit trail). Confirmability was ensured by describing what the study's phenomenon was, which control steering documents constituted data, what the study context was, and how data were analyzed (audit trail). Authenticity was ensured by all researchers using the study protocol (audit trail) and all research group members participating in the discussions of the results (member checking) [43, 44].

## Results

### Level I. National study programs

In the national study programs (*n* = 4), we found no content referring to actors and linked to context, actions, and relationships in the intended learning outcomes.

### Level II. Local program syllabuses

Of the 48 approved local program syllabuses, 7 (15%) included nine local intended learning outcomes of content referring to actors and linked to context, actions, and relationships. For example, one local program syllabus in medicine had the local intended learning outcome, ‘To use a patient-centered approach in clinical work,’ one local study program in occupational therapy had the local intended learning outcome, ‘To apply person-centered and reflective approach,’ and one local study program in nursing used the local intended learning outcome, ‘Have the competence to implement PCC.’ Of the 799 local course syllabuses identified in the local program syllabuses (*n* = 48), 8 (1%) had course titles referring to actors and linked to context, actions, and relationships. All identified course titles were in the nursing program. For example, two HEIs in nursing used the titles, ‘Person-centered care for mental illness’ and ‘Person-centered nursing, caring approach, and communication.’

### Level III. Local course syllabuses

Of the 799 local course syllabuses, 101 (13%) included 142 intended learning outcomes with content referring to actors and linked to context, actions, and relationships. For example, one of the intended learning outcomes in medical education was given as, ‘Using patient-centered conversation methodology, initiate the conversation and clarify the reason for the visit, including thought, concern, and desire.’ Another example, from the physiotherapist program, was, ‘Reflect on the importance of good communication and collaboration for effective person-centered care.’

Fourteen of the 48 HEIs (29%) did not have any content referring to actors and linked to context, actions, and relationships in their intended learning outcomes (Table 2). In addition, across the 48 HEIs, there was a difference when content referring to actors and linked to context, actions, and relationships in the intended learning outcome was introduced and taught (Table 2). In the national study programs in medicine, HEIs (*n* = 7) had most intended learning outcomes with content referring to actors and linked to context, actions, and relationships in semesters 4, 5, and 6 (each 17%). Furthermore, this study program had 4% intended learning outcomes with content referring to actors and linked to context, action, and relationship in semesters 7, 8, 9, and 11. However, HEIs of nursing (*n* = 25) had most intended learning outcomes with content referring to actors and linked to context, action and relationship (23.5%) in the first semester. HEIs of occupational therapy (*n* = 8) had most of their intended learning outcomes with content referring to actors and linked to context, actions, and relationships (33.3%) in semester 4 and HEIs of physiotherapy (*n* = 8) in semesters 4 and 6 (each 30%). Overall, the HEIs (*n* = 48) had most of their intended learning outcomes with content referring to actors and linked to context, actions and relationship in the sixth semester (28.2%).

**Table 2:** Higher education institutions and their content referring to actors and linked to context, actions, and relationships in their intended learning outcomes by semester

Total number of national study program sites	Total number of higher education institutions with no intended learning outcomes or content referring to actors and linked to context, actions, and relationships	Total number of included learning outcomes for each study site distributed over semesters					
		**1**	**2**	**3**	**4**	**5**	**6**
**Medicine (*n* = 7)**	1	2	3	4	2	4	4
	(14.3%)	(9%)	(13%)	(17%)	(9%)	(17%)	(17%)
**Nursing (*n* = 25)**	8	20	17	11	5	16	16
	(32%)	(23.5%)	(20%)	(12.9%)	(5.8%)	(18.8%)	(18.8%)
**Occupational therapy (*n* = 8)**	2	1	2	6	8	2	5
	(25%)	(4.1%)	(8.3%)	(25%)	(33.3%)	(8.3%)	(20.8%)
**Physio-therapy (*n* = 8)**	3	-	1	2	3	1	3
	(37.5%)		(10%)	(20%)	(30%)	(10%)	(30%)
**Total (*n* = 48)**	**14**						
	**(29%)**						
**Total intended learning outcomes (*n* = 138)**		**23**	**23**	**23**	**18**	**23**	**28**
		**(16.6%)**	**(16.6%)**	**(16.6%)**	**(13%)**	**(16.6%)**	**(20.2%)**

**Fig. 1:**
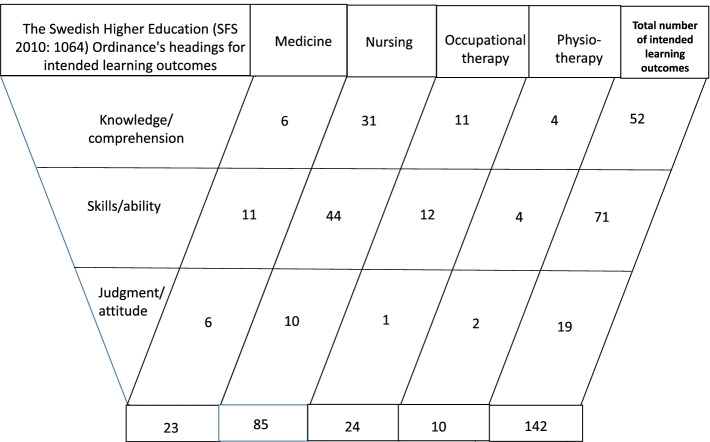
Schematic overview of the intended learning outcomes containing content referring to actors and linked to context, actions, and relationships in the local course syllabuses (level III) of the four national study programs (level I)

Of the 142 intended learning outcomes, 22 in nursing, 8 in occupational therapy, and 6 in physiotherapy needed to be assessed against Bloom’s taxonomy [39]. A total of 52 intended learning outcomes were connected to knowledge/comprehensibility. For example, in a medicine program, one was given as, ‘Describe the different parts of a person-centered patient–doctor conversation.’ A total of 71 intended learning outcomes were distributed under skills/ability. In the nursing program, one example was, ‘To prepare and conduct conversations with the individual and relatives in difficult and vulnerable situations based on a person-centered approach.’ Another intended learning outcome was in the physiotherapy program: ‘Be able to explain what person/family/child centering means in physiotherapeutic intervention.’ Finally, there were 19 intended learning outcomes distributed under judgment/attitude (Fig. 1). For example, in occupational therapy, an intended learning outcome was, ‘To further develop knowledge, skills, and values in applying occupational therapy based on a client-centered approach in collaboration with the client, relatives, and other professional groups involved.’

**Fig. 2:**
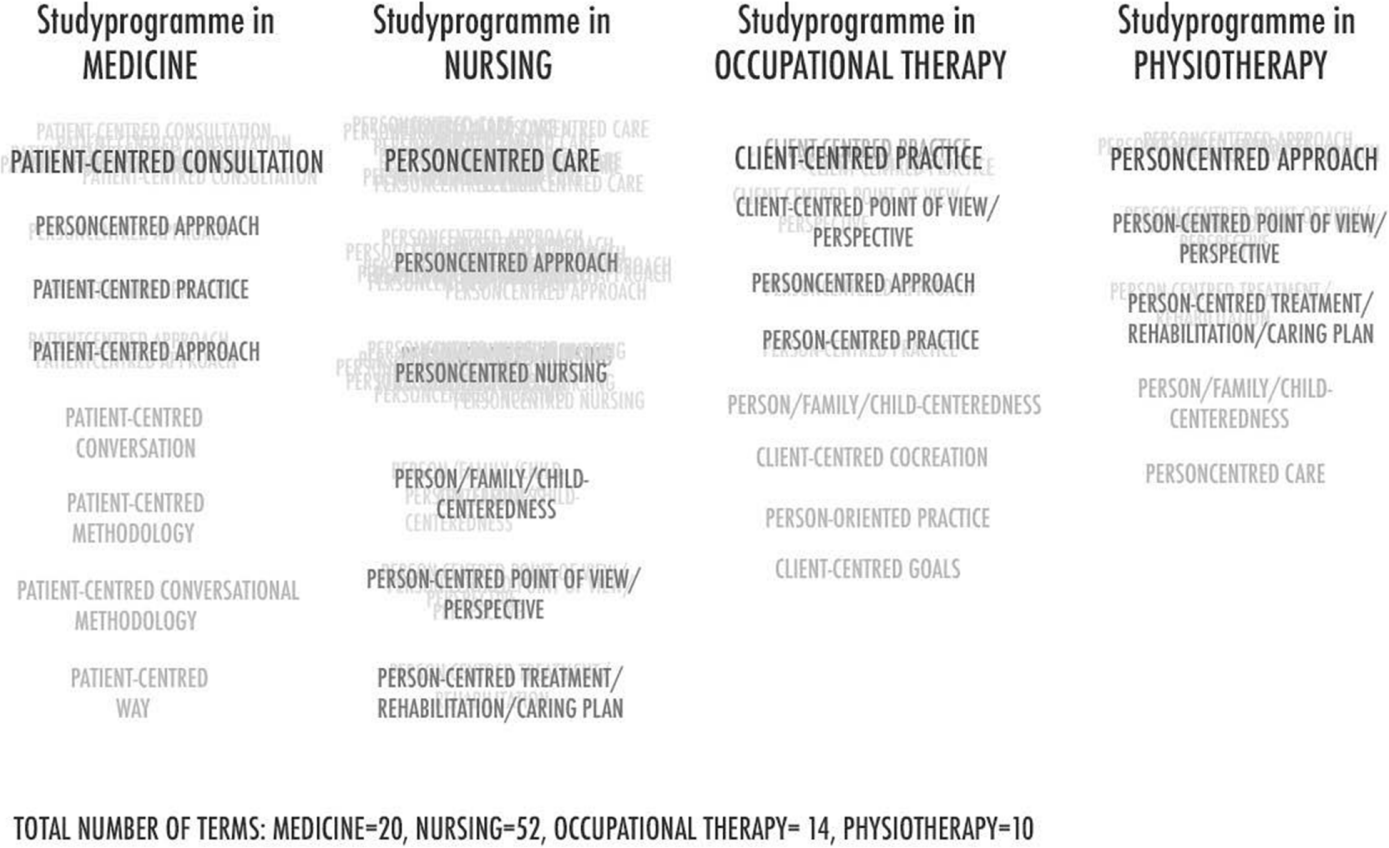
Overview of terms connected to content referring to actors and linked to context, actions, and relationships in the intended learning outcomes

Twenty-one terms connected to content referring to actors and linked to context, actions, and relationships were found in the intended learning outcomes. These 21 terms were used 96 times in the 142 identified outcomes. The most frequently used terms in the nursing study program were ‘person-centered approach’ (23 times) and ‘person-centered care’ (23 times). The most common terms in the medical study program were ‘patient-centered consultation’ (4 times), whereas the physiotherapy study program used ‘person-centered approach’ (2 times), ‘person-centered care’ (2 times), and ‘person-centered goal’ (2 times). In the occupational therapy study program, the term ‘client-centered practice’ (5 times) was most commonly used (Fig. 2.)

## Discussion

This study aimed to explore the PCC content in four Swedish national study programs in medicine, nursing, occupational therapy, and physiotherapy. No content referring to a person, patient, or client and linked to context, actions, and relationships was found in the level I steering documents but mainly in the level III documentation. In addition, there was an uneven representation and distribution of content referring to a person, patient, or client and linked to context, actions, and relationships between and within programs.

We identified local intended learning outcomes with PCC in three of the four national study programs at level II, and all examined national study programs had intended learning outcomes with PCC in their local course syllabuses (level III). The implication is that changes in the four Swedish national study programs are driven more often by the university lecturer, given that the local course syllabuses (level III; *n* = 101) contained more PCC references than the national study program (level I; *n* = 0) and local program syllabuses (level II; *n* = 7). On the one hand, our results could be interpreted as a lack of governance from authorities and leading politicians regarding the national study program. There is also a lack of governance of the faculties and departments at the universities when it comes to the local program syllabuses. On the other hand, the results could be seen as a good example of an implementation process driven by a bottom-up process in which the local teachers are the ones taking lead on the change.

In this study, we did not explore study guidelines, but it is not unreasonable to assume, as other studies have shown [24, 25], that the identified terms regarding PCC used in Swedish national study programs are inconsistently described. Bowden [27] pointed out that documents are rarely developed for research and, therefore, often contain few detailed descriptions. To gain a deeper understanding of the assumptions behind PCC, supplementing with other data, such as interviews, is recommended [27]. For this reason, we have started an interview study with program directors to obtain a better understanding of factors that promote or enable implementation of PCC in the four national study programs.

In this study, 21 different terms were used in reference to PCC, which is in line with earlier results [25]. The most commonly used terms in the present study were ‘person-centered approach’ and ‘person-centered care.’ In this study, we used a broad conceptual definition of PCC because we wanted to apply an inclusive approach. A problem identified by Sharma et al. [36] in 2015 was the lack of a universal definition. However, they identified several common components in the examined terms. Today, there is a European standard that describes minimal patient involvement in PCC [12] and is recommended as a tool for planning, implementing, and evaluating PCC in clinical practice and research [45, 46]. Based on our results demonstrating a large diversity of terms related to PCC, the standard could also be used for clarity and to plan, implement, and evaluate pedagogical and educational initiatives.

The results also show that most intended learning outcomes in the local course syllabuses (level III) could be classified within the skills/ability heading. It is reasonable that HEIs promote skills/ability if person-centered ethics [47] is the theoretical starting point. This ethical premise rewards actions directed at other humans (i.e., a form of applied ethics) [9]. However, 25% (36/142) of the local intended learning outcomes did not clearly state the level of knowledge that they reference. This lack is problematic because learning needs to be transparent so that the student knows what they know after completing a course or education program. Moreover, the content of a course should be able to communicate to the surrounding community. According to the Swedish Higher Education Act [29], ‘[T]he mandate of higher education shall include third stream activities and the provision of their activities, as well as ensuring that benefit is derived from their research findings.’

The uneven representation and distribution of PCC between and within programs calls for a consistent implementation strategy. Implementation requires that several actors from different societal systems work together, take a unified approach, and mutually drive the change forward [6, 19, 20]. Educational institutions are important facilitators by educating the next generation of professionals in PCC [6]. Therefore, it would be of interest to apply a strategy for implementation, and one available framework that can guide such implementation may be the ADDIE model [48]. The implementation of PCC is ongoing in the health care and social care sectors, but educational bodies have not yet been included in the strategy. Organizational culture was previously suggested to be regarded as an essential starting point before any change is implemented [49].

### Limitations

Many factors affect the quality of research when reviewing documents [27], and this study is no exception. Here, we explored documents, such as national study programs, local program syllabuses, and local course syllabuses, and conducted the study as a single look at these documents at a single point in time. Thus, it is possible that the included documents have been revised since the study was conducted. The explored documents also describe only the overall content of a program or course and contain few details, which can contribute to misinterpretations. Another limitation is that we only used documents as a data source in this study. This means that the result risks offering a one-sided and unvarnished picture of the content of the various documents. Another limitation is that we needed to assess 36 of 142 intended learning outcome levels of knowledge using Bloom’s taxonomy [39], so it is possible that we assessed them differently from what their developers intended.

## Conclusion

**Fig. 3:**
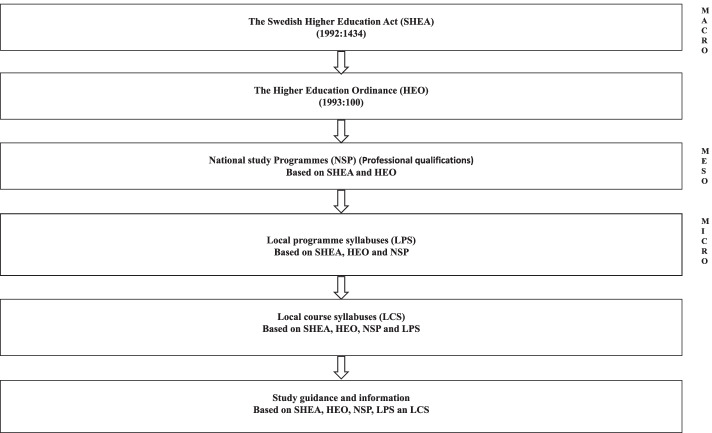
Overview of how Swedish higher education is governed

The change towards more PCC within the educational system is driven by local course leaders and teachers. Most PCC content found within the study programs was at a local level in intended course-learning outcomes. Increasing the inclusion of PCC instruction within and between the national study programs, local program syllabuses, and local course syllabuses requires action from politicians, authorities, faculty, and departments of higher education (Fig. 3). There is a need to further explore health care professionals’ education programs. More specifically, the content of study guides, offered learning activities, examination forms, and literature choices needs to be studied in detail. Furthermore, interviews with students and teachers will help us understand their learning of PCC in the context of higher education. There is also a need to support faculties to develop their knowledge and skills by offering in-service education to improve their practices regarding PCC.

## Data Availability

The datasets generated and/or analyzed during the current study are not publicly available due to the documents being written in the Swedish language (Fig. 3), but are available from the corresponding author on reasonable request.

## Abbreviations

HEC: Higher education credits
HEI: Higher education institution
PCC: Person-centered care
